# Interplay between heritability of smoking and environmental conditions? A comparison of two birth cohorts

**DOI:** 10.1186/1471-2458-11-316

**Published:** 2011-05-14

**Authors:** Jacqueline M Vink, Dorret I Boomsma

**Affiliations:** 1 Department of Biological Psychology, VU University, Amsterdam, The Netherlands; 2 Neuroscience Campus Amsterdam, Amsterdam, The Netherlands

## Abstract

**Background:**

Attitudes and policy towards smoking changed over the past years in many countries including the Netherlands. Generally, this led to a decrease in smoking prevalence. As demonstrated in twin and family studies, individual differences in smoking behavior are partly influenced by genetic factors. We explore whether the current change in environmental conditions has influenced the genetic architecture of smoking. This would constitute evidence for Gene × Environment (G×E) interaction.

**Methods:**

Data on smoking were available from 2 cohorts of young adult twins (18-25 year) registered with the Netherlands Twin Register. The first cohort completed a survey in 1993-1995 (n = 2669) and the second in 2009-2010 (n = 2339). Prevalence and genetic architecture of smoking were compared across cohorts using structural equation models in MX.

**Results:**

Smoking prevalence decreased from 40-51% to 22-23% between 1993-1995 and 2009-2010. Genetic analyses, making use of the different genetic resemblance in monozygotic and dizygotic twins, showed that the heritability was the same in both cohorts.

**Conclusions:**

The change in policy and smoking attitudes that led to a decrease in prevalence of smoking did not change the heritability of smoking and thus no evidence was found for GxE interaction.

## Background

Smoking ranks high among public health problems in the world. In the past years worldwide media campaigns discourage smoking behavior and emphasize the negative health consequences of smoking. In a large study among high school students in the United States, the prevalence for ever smoked cigarettes did not change from 1991 to 1999, but declined between 1999 and 2009 [1]. A study in Switzerland showed a decrease from 1992 to 2007 in the prevalence of ever smokers in men but not in women [2]. The same phenomenon was observed in Finnish men: the prevalence of smoking decreased from 1972 to 2007. Among women, smoking increased throughout the years until 2002 but remained stable between 2002 and 2007 [3].

In the Netherlands smoking policies changed in the past years. Cigarette packs contain warnings about health consequences since 2002 and advertisement for smoking is forbidden since 2002/2003 (before 2003 it was already forbidden for television and radio but since then also in newspapers, magazines et cetera). Smoking is forbidden in public transportation and at work since 2004 and it is not allowed to smoke in the catering industry since 2008. In addition, media campaigns discourage adolescents to take up smoking and the price of cigarettes increased greatly. The prevalence of current smokers gradually declined from the nineties till now http://www.stivoro.nl.

Understanding the trends in the prevalence of cigarette smoking among adolescents and young adults enables policy makers to target prevention resources more effectively. In this paper, we compare the prevalence of smoking in young adult (18-25 year old) twins who participated in the longitudinal survey study of the Netherlands Twin Register (NTR) in 1993-1995 with the prevalence of smoking in young adult twins who participated in 2009-2010.

Twin data offer the unique opportunity to disentangle genetic and environmental influences. Previous studies based on data from the NTR have shown that smoking in is influenced both by shared environmental (51-56%) and by genetic factors (36-44%) [4-6]. The estimates for the importance of those factors are comparable with other twin studies worldwide [7-10]. A meta-analysis estimated that, for men and women, 37% and 55% of the variance in smoking initiation was due to genetic factors, and 49% and 24% due to shared environmental factors [11].

In this paper we look at differences in heritability estimates between two birth cohorts. Such differences could point to Gene × Environment (G×E) interaction, which is defined as a difference in the influence of genetic factors conditional on environmental exposure [12]. The importance of the social context may influence heritability in several ways. According to previous studies, three complementary mechanisms can help explain G×E interaction mechanisms [13,14]. Under a *social control *mechanism the social forces wash out the effects of genetic factors in tobacco use. Norms and other social forces restrict variability in phenotype of genetically diverse people. For example, genetic influences on substances use (smoking & alcohol) were low or nonexistent among those who were raised with a strong religious upbringing [15,16]. According to the *social trigger *model, genetic factors differentiate between individuals only in the presence of social pressures. For example, the genetic influences on daily smoking were significantly higher in high school students who attended schools in which the most popular students smoked the most. In this case, the pro-smoking norms serve as trigger for the genetic influence. In the first two models, shared behavioral expectations and corresponding sanctions cause differences in heritability by blocking or enabling genes to be expressed. In contrast, the *social push *model suggests that changes in social norms can affect the relevance of genetic influences by minimizing or maximizing "noise" that has the potential to overwhelm or hide the influences (non-causal). As large numbers of people (regardless of genetic makeup) begin smoking, there will be a tipping point in the distribution of smoking environments where entrée into smoking becomes a primarily social phenomenon; genetic vulnerable persons are no more likely to begin smoking than genetically invulnerable persons simply because of the predominant social popularity of smoking (overwhelming influence of social factors). This was observed in Swedish women born between 1910 and 1924: the prevalence of smoking was very low and heritability was 0. Both prevalence and heritability increased in later birth cohorts when smoking among women became more accepted [17].

In this paper, we explored whether a change in environmental conditions led to a change in the relative contribution of genetic factors to smoking. This would constitute evidence for GxE interaction. Data on smoking were available from two cohorts of young adult twins (18-25 year) registered with the Netherlands Twin Register (NTR). The first cohort completed a questionnaire in 1993-1995 (n = 2669) and the second cohort in 2009-2010 (n = 2339). Genetic analyses, making use of the different genetic resemblance in Monozygotic (MZ) and Dizygotic (DZ) twins, will be used to explore heritability as a function of environmental exposure (1993-95 versus 2009-10).

## Methods

### Sample and measures

Smoking data were collected in a longitudinal survey study of the Netherlands Twin Register (NTR). The NTR was established in 1987 and registers twins and their family members who volunteer to taking part in research studies [18]. Since 1991, every two to three years surveys are mailed to adolescent and adult twins and their family members. Surveys focus on health, lifestyle, demographics, psychopathology and personality. Previous studies have shown that the non-response bias in the NTR sample is rather small [19,20]. All the NTR-work with human subjects is reviewed by the Central Ethics Committee on Research Involving Human Subjects of the VU University Medical Center, Amsterdam, an Institutional Review Board (IRB) certified by the US Office of Human Research Protections (IRB number IRB-2991 under Federal wide Assurance-3703; IRB/institute code NTR 03-180).

To compare cohort effects over a 15-year time interval, data collected in surveys in 1993-1995 were compared to data which were collected in 2009-2010. Data on smoking were available for 2305 18-25 year old twins who participated in 1993. Additional data on smoking initiation were added from 364 18-25 year old twins who participated in 1995. This combined dataset consisted of data on 2669 twins: 415 MZ male (206 pairs), 363 DZ male (178 pairs), 658 MZ female (326 pairs), 462 DZ female (227 pairs) and 769 DZ opposite sex twins (377 pairs).

The survey of 2009-2010 is ongoing. The available sample 18-25 year old twins consisted of 296 MZ male (105 pairs), 238 DZ male (74 pairs), 703 MZ female (269 pairs), 444 DZ female (149 pairs) and 649 dizygotic opposite sex twins (166 pairs).

The same question on smoking initiation was asked in both surveys: "Did you ever smoke?" with answer categories no, a few times to try and yes. Answers were recoded into a dichotomous variable: yes versus no or a few times to try.

### Analyses

Testing and genetic analyses were carried out using structural equation modeling in the software package MX [21]. A threshold model was used [22], which assumes an underlying (latent) liability to a categorical variable such as smoking. This liability is the sum of the effects of many genetic and environmental factors. It has a normal distribution with a mean of zero and unit variance. A threshold, based on the prevalence of smoking in the population divides the distribution into "affected" and "unaffected" individuals. A test for equality of thresholds between groups amounts to testing for equality of prevalence between e.g. cohorts or men and women. The resemblances between twins for the liability to smoking were summarized with tetrachoric twin correlations. Based on the correlations a genetic model can be fitted to the data to estimate the contribution of additive genetic factors (A), common environment (C) and unique environmental (E). The total variance of the liability was constrained to be 1 for men and women. Additive genetic effects are the same for MZ twins because they are genetically (nearly) identical. DZ twins however, share only 50% of their segregating genes. Common environmental effects are thought to be the same for MZ twins and DZ twins, based on the assumption that monozygotic twins do not share more environments with each other than DZ twins [23].

To investigate GxE, we tested whether the relative contributions of A, C and/or E were significantly different in the two cohorts. In addition, quantitative and qualitative sex differences were assessed. To test for quantitative sex differences we tested if the contributions of A, C and/or E differed between men and women. Finally, we assessed qualitative sex differences in the contribution of common environmental factors by specifying a free parameter in DOS twins that represents the correlation between the common environment of men and women (Rcdos). If Rcdos equals 1, this indicates that qualitative sex differences are not detected.

In the full ACE model (model 1) the influence of A, C and E factors was estimated separately for men and women and for both cohorts. The shared environmental correlation (Rcdos) in DZ twins of opposite sex (DOS) was estimated as a free parameter (in both cohorts). In model 2 the qualitative sex differences are tested (Rcdos = 1) while in model 3 to 5 the cohort differences for A, C and E were tested within sex. In model 6, quantitative sex differences were tested by equalizing the estimates for A, C and E for men and women. In models 7 and 8, A and C are dropped respectively.

Modeling was done on the raw data, using full-information maximum likelihood. This gives a likelihood (LL) for each model that is fitted to the data. Twice the difference between two nested models has a chi-square distribution with the degrees of freedom (df) equal to the difference in df between the two models. A p-value of 0.01 was used to test if a less complex sub model explained the data significantly worse than the more complex model.

## Results

A noteworthy decrease in smoking prevalence was observed from 1993-1995 to 2009-2010, in both sexes and in all zygosity groups (P < .001), as may be seen in Table 1. In the 1993-1995 cohort around 50% of the man and 40% of the women had initiated smoking while in the 2009-2010-cohort those percentages decreased to around 20% in both men and women. The prevalence of smoking was lower in MZ twins compared to DZ twins in both sexes and both cohorts (p < .001).

**Table 1:** Prevalence of smoking initiation in 18-25 year olds in 1993-1995 and 2009-2010

	18-25 year old twins in 1993-1995			18-25 year old twins in 2009-2010		
	**N total**	**N ever** **smoked**	**% ever** **smoked**	**N total**	**N ever** **smoked**	**% ever** **smoked**

**Men:**						

MZ	415	190	45.8%	301	44	14.6%
DZ ss	363	184	50.7%	239	69	28.9%
DZ os	384	218	56.8%	228	62	27.2%
Total	1162	592	50.9%	768	175	22.8%

**Women:**						

MZ	658	224	34.0%	728	128	17.6%
DZ ss	462	195	42.2%	445	122	27.4%
DZ os	385	176	45.7%	400	102	25.5%
Total	1505	595	39,5%	1573	352	22.4%

Tetrachoric twin correlations are shown in Table 2. Based on the differences in prevalence, different thresholds were retained for men and women and for monozygotic and dizygotic twins (4 different thresholds). In both cohorts, MZ correlations were higher than DZ correlations suggesting that genetic factors play a role in smoking initiation. The DZ correlations were higher than half the MZ correlations which suggest that shared environmental factors are also involved.

**Table 2:** Tetrachoric twin correlations (r) with 95% confidence interval (95%CI) for 18-25 year old twins from two birth cohorts

	1993-1995		2009-2010		Combined (1993-1995 & 2009-2010)	
	**r**	**95%CI**	**r**	**95%CI**	**r**	**95%CI**

MZM	.82	.70-.90	.75	.43 - .91	.80	.70 - .88
DZM	.51	.30-.67	.37	.04 - .72	.47	.29 - .63
MZF	.77	.66-.85	.80	.65 - .89	.78	.70 - .85
DZF	.69	.55 -.81	.66	.44 - .81	.67	.55 - .77
DOS	.40	.26 - .54	.34	.11 - .54	.38	.25 - .49

Heritability estimates are shown in Table 3. In both cohorts estimates were very similar.

**Table 3:** Genetic architecture of smoking behavior

	1993-1995		2009-2010		Combined (1993-1995 & 2009-2010)	
	**Estimate**	**95% CI**	**Estimate**	**95% CI**	**Estimate**	**95% CI**

**Man:**						

Genetic factors	.62	.23-.86	.67	.00 - .90	.66	.29 - .86
Shared environment	.19	.01-.53	.06	.00 - .66	.14	.00 - .46
Unique environment	.18	.10-.30	.26	.09 - .57	.20	.12 - .30

**Woman:**						

Genetic factors	.15	.00-.49	.34	.00 - .84	.21	.00 - .49
Shared environment	.62	.32-.80	.46	.00 - .79	.57	.32 - .77
Unique environment	.23	.15-.33	.20	.10 - .34	.22	.15 - .30

Estimates for the contribution of additive genetic, common environmental and unique environmental factors to the variance in smoking in 18-25 year old twins in 1993-1995 and 2009-2010 and in the combined sample (full model)

The correlation between the common environment of men and women (Rcdos) could be constrained at 1 (Table 4, model 2) indication no qualitative sex differences on smoking. Constraining the estimates for the contributions of genetic, common and unique environmental factors over the 2 cohorts did not deteriorate the fit of the model (Table 4, model 3 for men, model 4 for women, model 5 for men and women simultaneously), indicating that the estimates were not significantly different between the two cohorts. Based on the combined data from both cohorts 55% (95%CI: 36% - 75%) of the total variance in liability to smoking is explained by genes, 23% (95%CI: 7% - 39%) by shared environmental factors and the remaining variance of 21% (95%CI: 16% - 28%) is explained by unique environmental factors.

**Table 4:** Comparison of ACE models in both birth cohorts

	Compare cohorts	-2 LL	Df	Versus	**Χ**^**2**^	Δ df	p
1	Full ACE	5676.915	4990				
2	Constrain Rcdos at 1	5677.127	4992	1	0.212	2	0.8994
3	EQ ACE for men over cohorts	5677.825	4995	2	0.698	3	0.8737
4	EQ ACE for women over cohorts	5677.692	4995	2	0.565	3	0.9044
5	EQ ACE for both sexes over cohorts	5678.229	4998	2	1.102	6	0.9814
**6**	**No sex differences in contributions of A,C,E**	**5688.117**	**5001**	**5**	**9.888**	**3**	**0.0195**
7	Drop A	5718.180	5002	6	39.951	1	< 0.0001
8	Drop C	5695.529	5002	6	7,412	1	0.0064

Full ACE: the influence of A, C and E factors is estimated separately for men and women and for both cohorts. The shared environmental correlation (Rc) in DZ twins of opposite sex (DOS) is estimated as a free parameter. Model 2: test for qualitative sex differences (Rcdos is fixed at 1). Model 3: ACE in cohort 1993-1995 is equalized to ACE in cohort 2009-2010 for men. Model 4: ACE in cohort 1993-1995 is equalized to ACE in cohort 2009-2010 for women. Model 5: ACE in cohort 1993-1995 is equalized to ACE in cohort 2009-2010 for men and women simultaneously. Model 6: ACE for men is equalized to ACE for women. Model 7: Additive genetics effects (A) are dropped. Model 8: Shared environmental influences (C) are dropped

There was a trend (see Table 3) for heritability to be higher in men (62%-67%) than in women (15%-34%) while shared environmental influences were higher in women (.46-.62%) compared to men (.06%-19%). The test for sex differences in the contribution of genetic and environmental factors showed a p-value of 0.0195 (Table 4, model 6). With a critical p-value of 0.01 we concluded that the sex differences were not significant. Dropping the genetic factors or the shared environmental factors from the model caused a significant deterioration (Table 4, respectively model 7 and 8) in goodness-of-fit.

## Discussion

This study explored the prevalence and heritability of smoking initiation in two birth cohorts of young adults (18-25 years) and tested for Genotype × Environment interaction by estimating heritability conditional on birth cohort. The first cohort was born in 1968-1977 and their smoking behavior was assessed in 1993-1995. The prevalence of smoking was 50.9% in men and 39.5% in women. The second cohort was born in 1984-1992 and participated in the longitudinal survey study of the Netherlands Twin Register in 2009-2010. In this cohort a substantially lower prevalence of smoking was seen in both sexes with a prevalence of 22.8% in men and 22.4% in women. Thus, significantly more young adults started smoking in 1993-1995 compared to subjects of the same age in 2009-2010. Those results probably reflect the change in smoking policy in the Netherlands, indicating that the discouragement of smoking is successful. The observed decrease is in line with other studies in the Netherlands (a decrease of smoking from 38% to 30% for man and from 30% to 26% for women, from 1991 to 2009, http://www.stivoro.nl) suggesting that the NTR sample is representative for the Dutch population. A decrease in the prevalence of smoking is also seen in other European countries (a decrease in prevalence in Swiss and Finnish men [2,3]) and in the United States (a decline of smoking initiation among high school students [1]).

We evaluated the genetic architecture of smoking in both cohorts and did not find changes in the heritability as a function of environmental exposure. There are different theories to explain GxE interaction mechanisms. Policies and rules with respect to smoking behavior have changed between 1993-1995 and 2009-2010. This changed the general attitude from smoking as acceptable behavior to antismoking (= social push), and could *increase *heritability since only genetic vulnerable subjects still smoke. On the other hand, when smoking is stigmatized and banned in public places, social forces affect smoking among most persons, both with and without genetic tendencies to smoke (= social control). These social controls will cause an actual drop in smoking among genetically motivated smokers and will *decrease *the genetic influence on smoking [13]. The fact that we did not observe any changes in the heritability of smoking between both cohorts suggests either that those two mechanisms (social control and social push) counterbalance each other or that there are no GxE interactions for smoking in this age group.

In a Swedish study, regular tobacco use was explored in 3 different birth cohorts: 1910-1924, 1925-1939, 1940-1958. For men, the prevalence (65%, 66%, 72%) and the heritability estimates (53%, 58%, 51%) all were stable across the 3 birth cohorts. In contrast, the prevalence in women increased over time, from 18% in 1920-1924 to 52% in 1940-1958. The heritability estimates also increased remarkably from 0% to 64%. Kendler et al. suggested that heritability increased in women because the social restrictions on tobacco use in women became more relaxed over time [17]. The birth cohorts in the Swedish study were much earlier than the cohorts participating in the NTR study. The different outcomes in men and women in the Swedish data are therefore not surprising.

Heath *et al *(1993) explored data on smoking initiation in three adult twin samples from Australia, Virginia and AARP. Data were broken down into 4 or 5 birth cohorts ranging from 1915-1939 to 1960-1967. Despite a marked change in the proportion of male (not female) respondents who reported ever having smoked, no evidence was found for cohort differences in genetic and environmental effects (no Genotype × Cohort interaction) for either men or women [24]. Those results are in line with the present study. Heath *et al*. suggest that the same variables, like personality traits of biological effects, have continued to determine which individuals will become smokers, but that in more recent years only individuals with more extreme values on those traits are becoming smokers.

In the studies of Kendler *et al *and Heath *et al*, birth cohort and age effects are intertwined. Kendler *et al *showed that 20% of the total variance in lifetime regular tobacco use was explained by age. In contrast, in our study we analysed data of two groups of twins from different birth cohorts both measured when they were between 18 and 25 years old.

A study from the USA explored the heritability for smoking onset in different states rather than for different birth cohorts (mean age 16.4 years, age range 12-21, n = 2060 twin- and sibling pairs from 31 states) and reported that the average rate of smoking onset varied significantly across states (overall, 45% of the respondents reported smoking onset) but the measure of genetic influence did not. In contrast, the genetic influence for daily smoking varied across states [25].

Although the samples described in the other studies have different backgrounds compared to ours, the results suggest that the heritability of smoking initiation is not influenced by changes in prevalence ([24,25] and present study) while for regular/daily smoking the heritability seems to vary with fluctuating prevalence [17,25].

Boardman suggested that genes for smoking initiation may be different from those associated with daily smoking. Initiation of smoking might be more heavily influenced by personality characteristics such as novelty seeking or impulsivity, whereas regular tobacco use is related to other mechanisms such as nicotine metabolism. In line with this theory, recent meta-analyses of genome-wide association studies for smoking phenotypes detected significant associations between number of cigarettes smoked per day and polymorphisms in nicotine metabolism genes while those associations were not detected for smoking initiation [26,27].

We found that the genetic architecture for smoking initiation did not differ as a function of environmental conditions. In the data of the NTR, we also observed a change in the prevalence of alcohol use over the past 15 years (an increase). The heritability for alcohol use (alcohol initiation, frequency and quantity) was stable over the cohorts (Geels LM, Bartels M, van Beijsterveldt CEM, van der Aa N, Vink JM, Boomsma DI: Trends in adolescent alcohol use: Effects of age, sex and cohort, submitted). Those results are in line with our results on smoking initiation and suggest that changes in environmental factors causing a change in prevalence not automatically lead to change in the genetic architecture.

Our full genetic model suggested higher heritability estimates for men than for women while shared environmental influences were higher in women than men. A formal test showed that a model without sex differences did not worsen the fit of the model significantly, based on a p-value threshold of 0.01 but this test was borderline significant (p = 0.0195). Other studies exploring sex differences in genetic architecture showed contrasting results. A Finnish study on smoking initiation (early, late, never) reports a higher influence of genetic factors in men compared to women [7] while a meta-analysis for smoking initiation (based on 6 studies) showed a higher heritability in women compared to men [11], suggesting that if sex differences in heritability exist, they probably are not large. We did not find sex-differences in heritability of smoking initiation in an adult NTR sample [4] with a mean age of 30.5 years. The cohorts in the present study consist of younger adults (18-25) and at this age the contribution of environmental influences could differ for men and women. However, in a previous study of twins registered with the NTR, the predictors of the uptake of smoking were similar in boys and girls [28].

## Conclusions

In summary, these results describe a remarkable decrease in smoking between 1993-1995 and 2009-2010, but no changes in the relative contribution of genetic and environmental factors. Variation in smoking in 18-25 year olds was explained by genetic (55%), shared environmental (23%) and unique environmental (21%) factors. Those results are in line with other twin studies, both in Dutch samples and worldwide [4,5,9,11,29-31]. Future studies should incorporate additional phenotypes to test whether heritability will change for other smoking related phenotypes, such as number of cigarettes smoked per day or nicotine dependence. Furthermore, this research should be extended to other age groups. We focused on young adult twins aged 18-25 years because adolescence and young adulthood is the critical period to start smoking and relatively few individuals will start smoking after the age of 25. Finally, future studies of smoking behavior including genome-wide association studies should not only include age and sex, but should also consider birth-year and/or year of assessment as important covariates to explain differences in prevalence.

## List of abbreviations

G×E: Gene × Environment; MZ: Monozygotic; DZ: Dizygotic; NTR: Netherlands Twin Register; A: genetic variance; C: common environmental variance; E: unique environmental variance

## Competing interests

The authors declare that they have no competing interests.

## Authors' contributions

JV carried out the statistical analyses and wrote the manuscript. DB is head of the Netherlands Twin Register, supervised the project and contributed to the manuscript. Both authors read and approved the final manuscript.

## Pre-publication history

The pre-publication history for this paper can be accessed here:

http://www.biomedcentral.com/1471-2458/11/316/prepub

## References

[B1] Center for Disease Control and Prevention Cigarette use among high school students - United States, 1991-2009 MMWR Morb Mortal Wkly Rep 2010 59 26 797 801 20613702

[B2] Marques-Vidal P Cerveira J Paccaud F Cornuz J Smoking trends in Switzerland, 1992-2007: a time for optimism? J Epidemiol Community Health 2010 10.1136/jech.2009.099424 20427551

[B3] Vartiainen E Laatikainen T Peltonen M Juolevi A Männistö S Sundvall J Jousilahti P Salomaa V Valsta L Puska P Thirty-five-year trends in cardiovascular risk factors in Finland Int J Epidemiol 2010 39 2 504 518 10.1093/ije/dyp330 19959603

[B4] Vink JM Willemsen G Boomsma DI Heritability of smoking initiation and nicotine dependence Behav Genet 2005 35 4 397 406 10.1007/s10519-004-1327-8 15971021

[B5] Vink JM Beem AL Posthuma D Neale MC Willemsen G Kendler KS Slagboom PE Boomsma DI Linkage analysis of smoking initiation and quantity in Dutch sibling pairs The Pharmacogenomics Journal 2004 4 274 282 10.1038/sj.tpj.6500255 15170444

[B6] Koopmans JR Slutske W Heath AC Neale MC Boomsma DI The genectics of smoking initiation and quantity smoked in Dutch adolescent and young adult twins Behavior Genetics 1999 29 6 383 393 10.1023/A:1021618719735 10857244

[B7] Broms U Silventoinen K Madden PAF Heath AC Kaprio J Genetic architecture of smoking behavior: a study of Finnish adult twins Hum Genet 2006 9 64 72 10.1375/183242706776403046 16611469

[B8] Heath AC Madden PAF MArtin NG Statisitcal methods in genetic research on smoking Statistical methods in medical research 1998 7 165 186 10.1191/096228098676685170 9654640

[B9] Kendler KS Neale MC Sullivan P Corey LA Gardner CO Prescott CA A population-based twin study in women of smoking initiation and nicotine dependence Psychological Medicine 1999 29 299 308 10.1017/S0033291798008022 10218922

[B10] Maes H Neale M Kendler K Martin N Heath A Eaves L Genetic and cultural transmission of smoking initiation: an extended twin kinship model Behavior Genetics 2006 36 6 795 808 10.1007/s10519-006-9085-4 16810566

[B11] Li MD Cheng R Ma JZ Swan GE A meta-analysis of estimated and environmental effects on smoking behavior in male and female adult twins Addiction 2003 98 1 23 31 10.1046/j.1360-0443.2003.00295.x 12492752

[B12] Boomsma DI Martin NG H D'haenen JAdBaPW Gene-Environment Interactions Biological Psychiatry 2002 John Wiley & Sons 181 187

[B13] Boardman JD Blalock CL Pampel FC Trends in Genetic Influences of Smoking Journal of Health and Social Behavior 2010 51 108 123 10.1177/0022146509361195 20420298 PMC3158572

[B14] Shanahan MJ Hofer SM Social context in gene-environment interactions: retrospect and prospect The Journals of Gerontology 2005 60B 65 76 10.1093/geronb/60.special_issue_1.65 15863711

[B15] Koopmans JR Slutske WS Van Baal GC Boomsma DI The influence of religion on alcohol use initiation: evidence for genotype X environment interaction Behav Genet 1999 29 6 445 453 10.1023/A:1021679005623 10857249

[B16] Timberlake DS Rhee SH Haberstick BC Hopfer CJ Ehringer MA Lessem JM Smolen A Hewitt JK The moderating effects of religiosity on the genetic and environmental determinants of smoking initiation Nicotine Tob Res 2006 8 1 123 133 10.1080/14622200500432054 16497606

[B17] Kendler KS Thornton LM Pedersen NL Tobacco consumption in Swedish twins reared apart and reared together Arch Gen Psychiatry 2000 57 886 892 10.1001/archpsyc.57.9.886 10986552

[B18] Boomsma DI de Geus EJC Vink JM Stubbe JH Distel MA Hottenga JJ Posthuma D van Beijstervelt TC Hudziak JJ Bartels M Netherlands Twin Register: from twins to twin families Twin Research and Human Genetics 2006 9 6 849 857 10.1375/twin.9.6.849 17254420

[B19] Vink JM Boomsma DI A comparison of early and late respondents in a twin-family survey study Twin Res Hum Genet 2008 11 2 165 173 10.1375/twin.11.2.165 18361718

[B20] Distel MA Ligthart L Willemsen G Nyholt DR Trull TJ Boomsma DI Personality, health and lifestyle in a questionnaire family study: a comparison between highly cooperative and less cooperative families Twin Res Hum Genet 2007 10 2 348 353 10.1375/twin.10.2.348 17564524

[B21] Neale MC Boker SM Xie G Maes HH Mx: Statistical Modeling 2006 Richmond, Virginia: Department of Psychiatry: Virginia Commonwealth University

[B22] Falconer DS Mackay TFC Treshold Characters (chapter 18) Quantitative Genetics 1996 Essex: Longman Group Ltd

[B23] Boomsma DI Busjahn A Peltonen L Classical twin studies and beyond Nature reviews genetics 2002 3 872 882 10.1038/nrg932 12415317

[B24] Heath AC Cates R Martin NG Meyer J Hewitt JK Neale MC Eaves LJ Genetic contribution to risk of smoking initiation: comparisons across birth cohorts and across cultures Journal of Substance Abuse 1993 5 221 246 10.1016/0899-3289(93)90065-J 8312729

[B25] Boardman JD State-Level Moderation of Genetic Tendencies to Smoke American Journal of Public Health 2009 99 3 10.2105/AJPH.2008.134932 PMC2661451 19150910

[B26] Tobacco-and-Genetics-Consortium Meta-analyses of genome-wide association studies implicate multiple loci for smoking behavior Nature Genetics 2010 42 5 441 447 10.1038/ng.571 20418890 PMC2914600

[B27] Thorgeirsson TE Gudbjartsson DF Surakka I Vink JM Amin N Sulem P consortium eaE Sequence variants at the CHRNB3-CHRNA6 and CYP2A6 loci influence smoking behavior Nature Genetics 2010 42 5 448 453 10.1038/ng.573 20418888 PMC3080600

[B28] Vink JM Willemsen G Engels RCME Boomsma DI Smoking status of parents, siblings and friends: predictors of regular smoking? Findings from a longitudinal twin-family study Twin Research 2003 6 3 209 217 10.1375/136905203765693861 12855070

[B29] Kaprio J Pulkkinen L Rose RJ Genetic and environmental factors in health-related behaviors: studies on finnish twins and twin families Twin Research and Human Genetics 2002 5 5 366 371 10.1375/twin.5.5.366 12537860

[B30] Heath AC Madden PAF Genetic influences on smoking behavior Behavior Genetic Approaches in Behavioral Medicine 1995 New York: Plenum Press 45 66

[B31] Maes HH Sullivan PF Bulik CM Neale MC Prescott CA Eaves LJ Kendler KS A twin study of genetic and environmental influences on tobacco initiation, regular tobacco use and nicotine dependence Psychol Med 2004 34 7 1251 1261 10.1017/S0033291704002405 15697051

